# A Co-Delivery System of Curcumin and p53 for Enhancing the Sensitivity of Drug-Resistant Ovarian Cancer Cells to Cisplatin

**DOI:** 10.3390/molecules25112621

**Published:** 2020-06-04

**Authors:** Xinli Guo, Zhou Fang, Min Zhang, Deyu Yang, Shuyue Wang, Kehai Liu

**Affiliations:** College of Food Science and Technology, Shanghai Ocean University, Shanghai 201306, China; gxllps@163.com (X.G.); fangzhou0117@163.com (Z.F.); zm20180908@163.com (M.Z.); YYYYYDY7@163.com (D.Y.); Shuyue_Wang@163.com (S.W.)

**Keywords:** ovarian cancer, drug resistance, polyethyleneimine, curcumin, p53, co-delivery system

## Abstract

In order to enhance the sensitivity of drug-resistant ovarian cancer cells to cisplatin (DDP), a co-delivery system was designed for simultaneous delivery of curcumin (CUR) and p53 DNA. Firstly, the bifunctional peptide K14 composed of tumor targeting peptide (tLyP-1) and nuclear localization signal (NLS) was synthesized. A nonviral carrier (PEI-K14) was synthesized by cross-linking low molecular weight polyethyleneimine (PEI) with K14. Then, CUR was coupled to PEI-K14 by matrix metalloproteinase 9 (MMP9)-cleavable peptide to prepare CUR-PEI-K14. A co-delivery system, named CUR-PEI-K14/p53, was obtained by CUR-PEI-K14 and p53 self-assembly. Furthermore, the physicochemical properties and gene transfection efficiency were evaluated. Finally, ovarian cancer cisplatin-resistant (SKOV3-DDP) cells were selected to evaluate the effect of CUR-PEI-K14/p53 on enhancing the sensitivity of drug-resistant cells to DDP. The CUR-PEI-K14/DNA complexes appeared uniformly dispersed and spherical. The particle size was around 20–150 nm and the zeta potential was around 18–37 mV. It had good stability, high transfection efficiency, and low cytotoxicity. CUR-PEI-K14/p53 could significantly increase the sensitivity of SKOV3-DDP cells to DDP, and this effect was better as combined with DDP. The sensitizing effect might be related to the upregulation of p53 messenger RNA (mRNA), the downregulation of P-glycoprotein (P-gp) mRNA, and the upregulation of BCL2-Associated X (bax) mRNA. CUR-PEI-K14/p53 can be used as an effective strategy to enhance the sensitivity of drug-resistant ovarian cancer cells to DDP.

## 1. Introduction

Ovarian cancer is a common cancer in women worldwide, and accounts for 5% of overall cancer deaths [1,2]. At present, platinum anticancer drugs such as cisplatin (DDP) and carboplatin are the main chemotherapy protocol for advanced ovarian cancer, but with the progress of treatment, the sensitivity of patients to platinum drugs was decreased, especially after the recurrence [3]. Although the therapeutic effect of advanced ovarian cancer was improved with an improved dosage regimen or in combination with other drugs, the overall five-year survival rate remained low [4]. The main limitation is the emergence of chemotherapy resistance.

The process of chemotherapy resistance of ovarian cancer is complex, during which, suppressor genes play an important role. The p53 is a common tumor suppressor and it is essential to maintain normal genomic stability. Wild-type p53 arrests the cell cycle in the first gap (G1) or second gap/metaphase (G2/M) phase, or initiates an apoptotic to repair DNA damage [5,6]. However, the mutant p53 does not have this effect. Studies showed that most patients with ovarian cancer had p53 mutations, and mutant p53 was proved to be associated with chemotherapy resistance in ovarian cancer [7,8,9,10]. Gu et al. found that the activation of p53 was a decisive factor in increasing the sensitivity of cells to DDP [11]. Therefore, the gene therapy of platinum-resistant ovarian cancer is mainly focused on p53. Gene therapy is to introduce exogenous target genes into the target cells, and with the gene expression, the cells acquire specific functions to achieve the therapeutic purpose. How to successfully deliver p53 to drug-resistant ovarian cancer cells is the key to effectively increase the sensitivity of cells to DDP. In addition, drug resistance has generally been associated with drug efflux [12]. P-glycoprotein (P-gp) is a membrane glycoprotein with the function of drug efflux pump. It relies on intracellular adenosine triphosphate (ATP) to expel a variety of cytotoxic drugs out of cells, increasing the resistance of tumor cells to drugs [13,14]. Therefore, how to repress the expression of P-gp is the main measure to increase the sensitivity of drug-resistant cells to DDP. Curcumin (CUR) is a natural polyphenol compound from *Curcuma longa L*., which has a wide range of biological activities including anti-inflammatory, anti-oxidant, anti-infection, and anti-cancer [15,16]. It interacts with a variety of target molecules, including transcription factors, receptors, kinases, growth factors, enzymes, and cytokines, and can interfere with multiple signaling pathways [17]. Studies showed that CUR could promote the expression of p53 and destroy the internal structure of mutant p53 [18,19]. In addition, it could downregulate the expression of P-gp [20,21].

From the above analysis, it can be seen that p53 and CUR can synergistically enhance the sensitivity of drug-resistant cells to chemotherapy. However, to better play the synergistic effect of p53 and CUR, a safe and efficient co-delivery system is needed, which is the basis for the success of synergistic drug resistance reversal.

The delivery of target gene is the bottleneck of gene therapy and the fundamental reason that affects its effectiveness [22]. At present, the biggest problem of p53 gene delivery is that it has not yet obtained an ideal transgenic vector. Transgenic vectors include viral vectors and nonviral vectors. Nonviral vectors are being developed rapidly for the use of gene delivery due to low immune response and high carrier capacity [23]. Among the nonviral vectors, polyethyleneimine (PEI) has gained increasing attention because of its strong buffering ability and strong ability to condense DNA and adhere to cell membranes. In addition, PEI contains a large number of cations that can condense DNA into nanoparticles to mediate transfection, protect DNA from enzymatic degradation, and facilitate cellular uptake and endolysosomal escape [24]. However, high cytotoxicity, poor in vivo targeting, and nuclear transport barriers limit its practical application [25,26].

Neuropilin receptor (NRP), one of the focused areas in cancer, was highly expressed in ovarian cancer cells and could be used as one of the markers of malignant tumors [27,28]. Studies showed that 95.7% of the cells were positive stain for NRP-related antigen by immunohistochemistry assay in human epithelial ovarian cancer [29]. It plays an important role in chemotherapy resistance, and may serve as a potential drug therapeutic target for ovarian cancer [30,31,32]. Peptide tLyp-1 with the sequence Cys-Gly-Asn-Lys-Arg-Thr-Arg (CGNKRTR) was a truncated form of LyP-1 and reported as a targeting ligand to the NRP with high affinity and specificity [33]. Roth reported that tLyP-1 could specifically target and bind to NRP and produce the effect of transvascular penetration and tumor membrane penetration [34]. On the other hand, the introduction of nuclear localization signal (NLS) sequence into tLyP-1 sequence can promote the nuclear delivery of the complex after entering the cells. It is very important for the complex to overcome the nuclear membrane barrier and improve the transfection efficiency. Talsma et al. confirmed that compared with unmodified PEI, the transfection efficiency of PEI modified with α1-NLS was improved by 16 times [35]. The macromolecular substances can be actively transported from the cytoplasm to the nucleus via the nuclear pore complex mediated by NLS [35,36,37]. At present, the most widely studied functional sequence of NLS is Lys-Lys-Lys-Arg-Lys (KKKRK) [36,38]. Therefore, in this study, NLS sequence (KKKRK) was introduced into tLyP-1 sequence (CGNKRTR) to synthesize a bifunctional peptide tLyP-1-NLS (sequence C-KKKRK-CGNKRTR-C, named K14), which could enhance the selectivity of the vector to ovarian cancer cells and improve the ability of nuclear delivery, thus increasing the transfection efficiency of DNA.

Furthermore, low-molecular-weight (LMW) PEI was cross-linked with K14 to form a high-molecular-weight (HMW) PEI derivative PEI-K14, which can ensure high transfection efficiency similar to that of HMW PEI. At the same time, PEI-K14 was still assembled by LMW PEI with low toxicity, which makes it greatly reduce the cytotoxicity. In addition, a large number of positive charges on the surface of PEI-K14 could form a stable nanocomplex with p53 by electrostatic adsorption.

The occurrence and metastasis of tumors must break through the extracellular matrix at the primary sites of tumors. Therefore, matrix metalloproteinases (MMPs), which can degrade matrix components, play an important role in the occurrence and progression of tumors, especially in invasion and metastasis [39]. MMPs are overexpressed in almost all human tumors. In all MMPs, the upregulation of MMP9 was considered to be related to the diagnosis and prognosis of cancer, which also provided a drug administration strategy through enzyme-triggered mechanism [40]. MMP9 exists outside the tumor cells, so the enzyme-triggered deformation of the carrier can occur outside the tumor cells without entering the cells. The peptide with the sequence of PLGIAG can be cleaved by MMP9 and used as an environment responsive block of nanocarrier [41]. In this study, CUR was conjugated with PEI-K14 through CPLGLAG block, which was broken by MMP9 enzymatic hydrolysis in the tumor extracellular matrix. CUR was released to play a role in reducing cell resistance.

**Figure 1:**
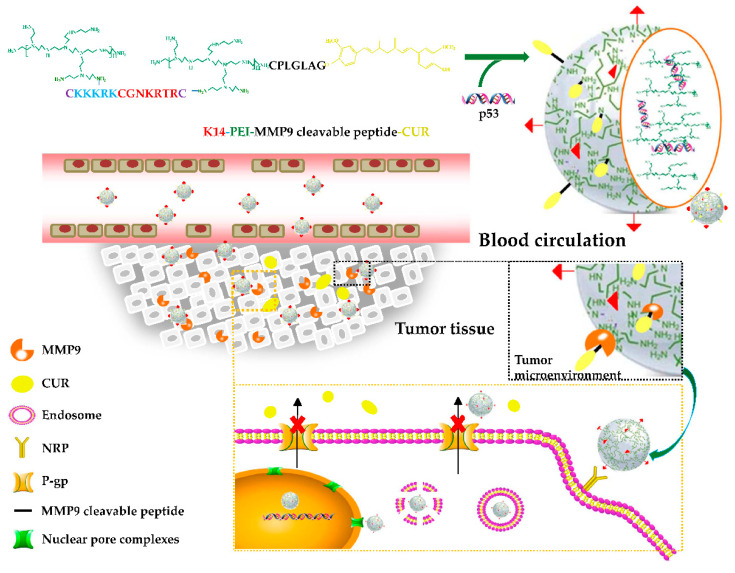
Preparation and delivery of the co-delivery system CUR-PEI-K14/p53.

In this study, the HMW PEI-K14 was prepared by cross-linking LMW PEI with a bifunctional peptide K14, and then coupled with CUR through CPLGIAG peptide, and at last self-assembled with p53 to form a co-delivery system CUR-PEI-K14/p53, so as to deliver CUR and p53 simultaneously, and synergistically enhance the sensitivity of ovarian cancer resistant cells to platinum chemotherapy. Figure 1 shows the preparation and delivery of CUR-PEI-K14/p53. The co-delivery system was actively targeted and concentrated in the tumor tissue through the tumor-targeting peptide tLyP-1 sequence in K14, and then the CPLGIAG peptide was enzymolyzed by MMP9, and CUR was released, downregulating the expression of P-gp. After the release of CUR, the PEI-K14/p53 complex entered tumor cells through endocytosis. The NLS sequence in K14 actively carried the complex into the nucleus, and p53 was released, replicated, and transcribed. CUR and p53 were co-delivered to the same tumor cells and increased the sensitivity of drug-resistant cells to DDP.

## 2. Results and Discussion

### 2.1. Synthesis and Characterization of CUR-PEI-K14

**Figure 2:**
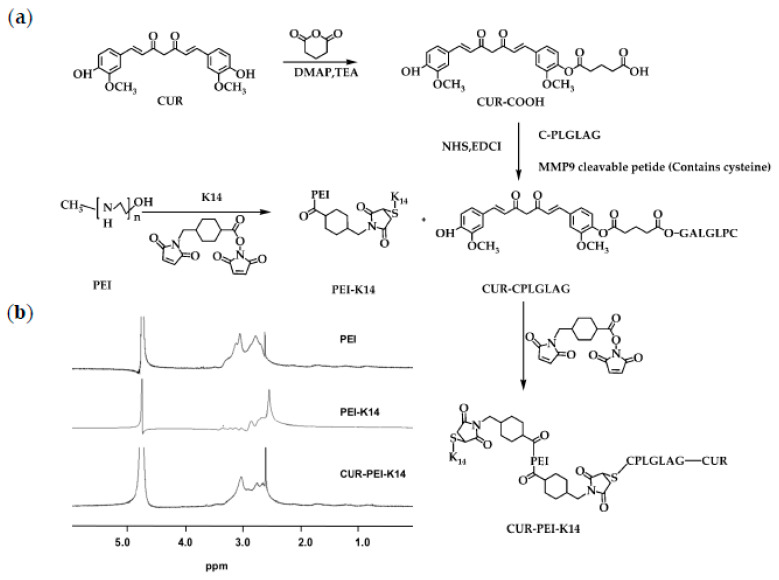
(**a**) Synthetic scheme of CUR-PEI-K14. (**b**) Representative ^1^H-NMR spectra of PEI, PEI-K14, and CUR-PEI-K14 in D_2_O at room temperature.

At first, K14 and CPLGLAG were synthesized using solid-phase method. Then, the bifunctional peptide K14 was used to cross-link PEI by 4-(*N*-Maleimidomethyl)cyclohexanecarboxylic acid *N*-hydroxysuccinimide ester (SMCC) to synthesize PEI-K14. After CUR-CPLGLAG was prepared, it was coupled with PEI-K14 via SMCC to obtain the target product CUR-PEI-K14, as shown in Figure 2a. The ^1^H-NMR spectra of PEI, PEI-K14, and CUR-PEI-K14 are shown in Figure 2b. The characteristic peak of PEI was 3.0 ppm, which was mainly generated by the -CH_2_CH_2_NH- repeat structure of PEI. In the ^1^H-NMR spectrum of PEI-K14, the characteristic peak at 3.0 ppm was leveled out due to the introduction of carbonyl groups in K14, indicating the presence of K14 in PEI-K14. Comparing the ^1^H-NMR spectra of PEI and PEI-K14, the characteristic peak of CUR-PEI-K14 was in a lower magnetic field, located at 2.8 ppm, which was caused by the electronic shielding effect of CUR-CPLGLAG attachment. All these data revealed that CUR-PEI-K14 was synthesized successfully.

### 2.2. Condensation of Plasmid DNA (pDNA) by CUR-PEI-K14/DNA

**Figure 3:**
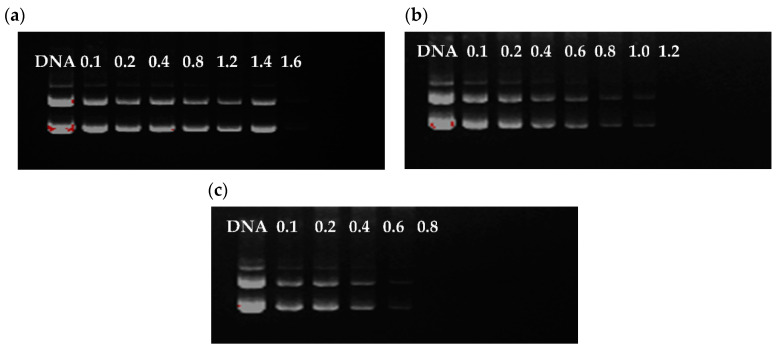
Agarose gel electrophoresis of plasmid DNA and (**a**) CUR-PEI-K14-1, (**b**) CUR-PEI-K14-3, and (**c**) CUR-PEI-K14-10 complexes at various *w*/*w* ratios.

The DNA condensation capacity of polymer is critical to the successful delivery of DNA. Figure 3 demonstrates that the DNA bands faded gradually and disappeared with the increase of weight/weight (*w*/*w)* ratio, indicating that the ability of CUR-PEI-K14 to condense DNA was enhanced. The migration of DNA was completely retarded when the *w*/*w* ratio of CUR-PEI-K14-1/DNA was 1.6. CUR-PEI-K14-3 and CUR-PEI-K14-10 had little free DNA escape at w/w ratios of 1.2 and 0.8, respectively, indicating that the condensation ability increased gradually with increasing K14/CUR-PEI ratio. This phenomenon might have been mediated by spatial effect of the attached K14, promoting the combination of CUR-PEI-K14 and DNA.

### 2.3. Particle Size, Zeta Potential, and Morphology of CUR-PEI-K14/DNA

DNA can be encapsulated in cationic polymers through electrostatic interactions [42]. Generally, spherical shape, appropriate particle size, and positive surface charge give polymers better transfection efficiency [22].

**Figure 4:**
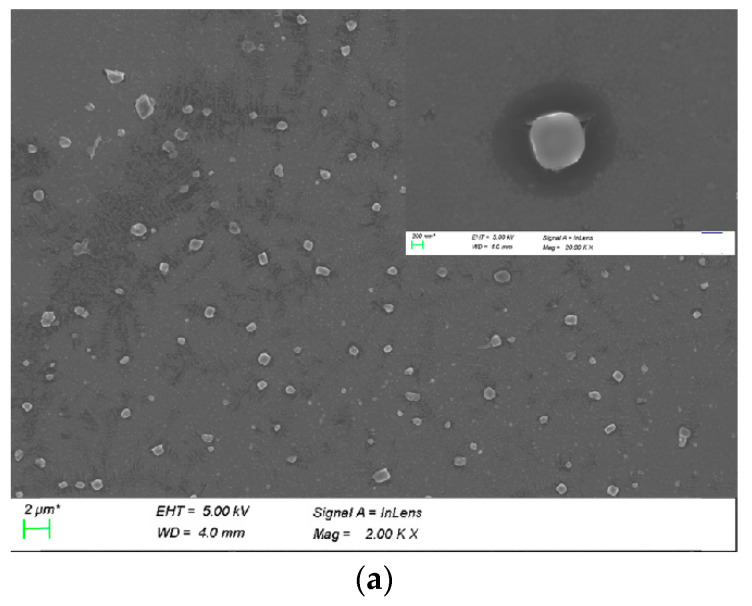

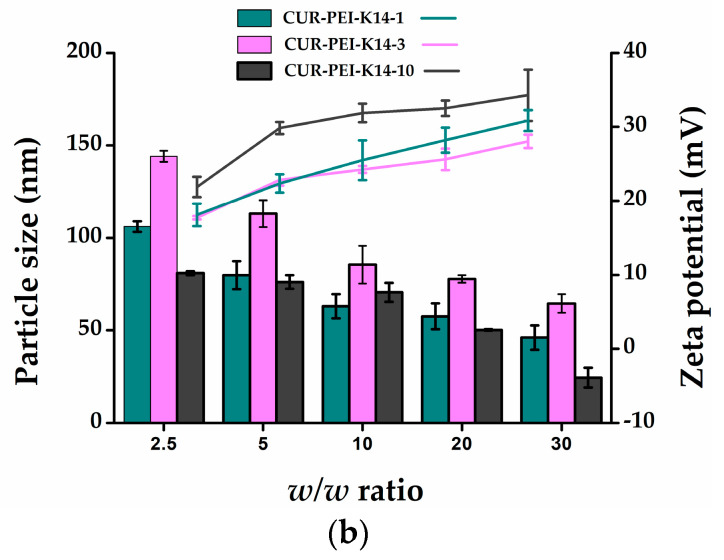
(**a**) SEM image of CUR-PEI-K14/DNA complexes. (**b**) Particle sizes (nm) and zeta potential (mV) of CUR-PEI-K14/DNA complexes at various *w*/*w* ratios.

Figure S1 shows that CUR-PEI-K14 was uniformly dispersed and stable at room temperature. After CUR-PEI-K14 condensed DNA, the complex turned into a compact and stable spherical nanoparticle, according to Figure 4a.

Figure 4b shows that the particle sizes of the CUR-PEI-K14/DNA ranged from 20 to 150 nm and the zeta potentials ranged from 18 to 37 mV in the *w*/*w* ratio range of 2.5–30. In addition, as the *w*/*w* ratio increased, the zeta potential gradually increased and the particle size gradually decreased, indicating that the ability to condense DNA was enhanced with the increase of the surface positive charge, which led to compact structure and smaller particle size.

### 2.4. Stability of CUR-PEI-K14/DNA Complexes

**Figure 5:**
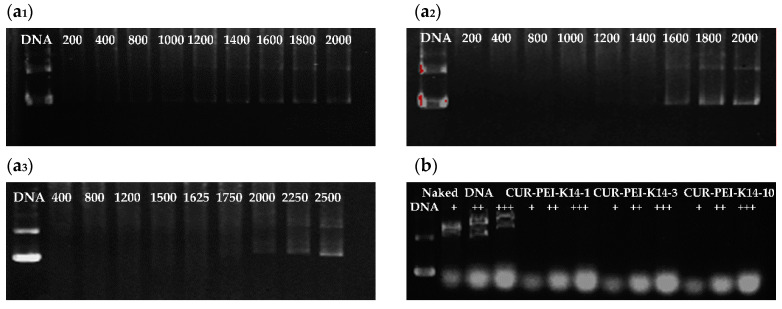
Protection of (**a_1_**) CUR-PEI-K14-1, (**a_2_**) CUR-PEI-K14-3, and (**a_3_**) CUR-PEI-K14-10 on pDNA from dissociation by sodium heparin at varying concentrations (μg/mL). Protection of (**b**) CUR-PEI-K14 on pDNA from dissociation by serum at varying concentrations. The “+” means the serum at concentration of 25%, “++” means the serum at concentration of 50%, and “+++” means the serum at concentration of 75%.

The components in the blood were complicated. After entering the body, the DNA complex might be dissociated by certain negatively charged inorganic salts and proteins. Therefore, in vivo stability of the CUR-PEI-K14/DNA complex was simulated and evaluated by incubation with heparin and serum in vitro. As illustrated in Figure 5a, CUR-PEI-K14-1/DNA, CUR-PEI-K14-3/DNA, and CUR-PEI-K14-10/DNA were dissociated by sodium heparin at concentrations of 1200, 1600, and 2000 μg/mL, respectively, indicating that CUR-PEI-K14/DNA could be resistant to dissociation of at least 1200 μg/mL sodium heparin. Furthermore, the anti-dissociation capacity of CUR-PEI-K14 was enhanced as the K14/CUR-PEI ratio increased.

In addition, it can be seen from Figure 5b that some new DNA bands appeared in the naked DNA group with the increase of serum concentration. This might be related to the degradation of DNA by serum. However, there was no new DNA band in CUR-PEI-K14 group. Therefore, CUR-PEI-K14/DNA could stably exist in serum. Taken together, these results can predict in vivo stability of the CUR-PEI-K14/DNA complex.

### 2.5. Gene Transfection In Vitro

**Figure 6:**
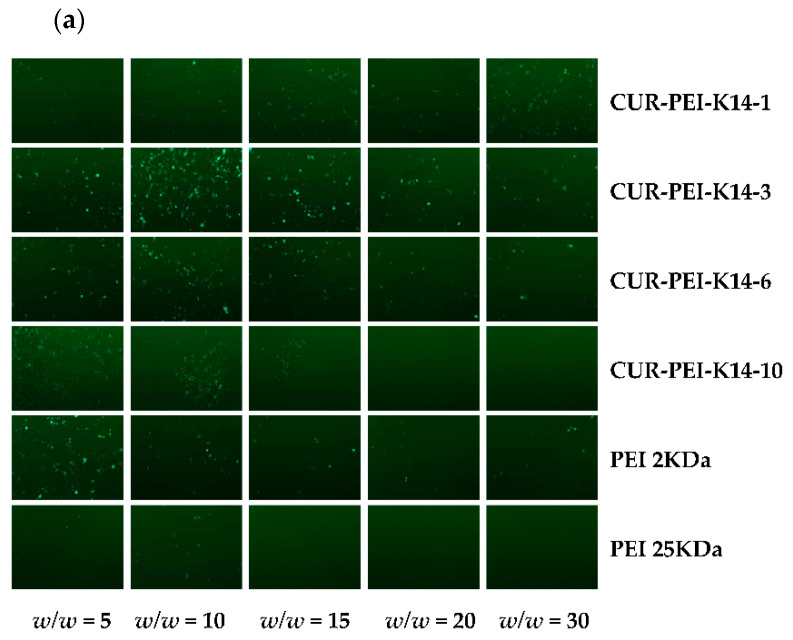

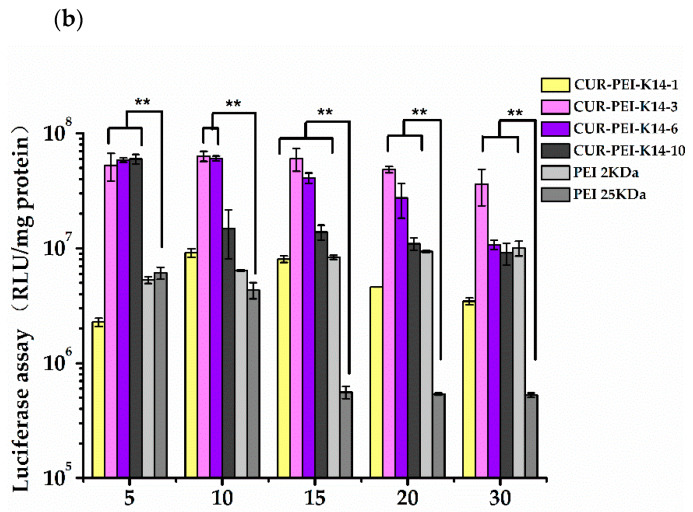
Transfection efficiency of (**a**) *pEGFP-N2* and (**b**) *pGL3-Control* in SKOV3-DDP cells by different polymer/DNA complexes at *w*/*w* ratios of 5, 10, 15, 20, and 30. Each data point represents the mean ± standard deviation (*n* = 3, ** *P* < 0.01). *pEGFP-N2* is a reporter gene that can be used to qualitative analyze transfection efficiency; *pGL3-Control* is a reporter gene that can be used to quantitatively analyze transfection efficiency.

It can be seen from Figure 6a that PEI 25 kDa/DNA showed obvious green fluorescent protein expression at *w*/*w* ratio of 5, but the transfection effect of complexes decreased significantly with the increase of *w*/*w* ratio. This phenomenon might have been mediated by high cytotoxicity of PEI 25,000 Da (25 kDa) in ovarian cancer cisplatin-resistant (SKOV3-DDP) cells. In comparison, PEI 2 kDa/DNA still had green fluorescent protein expression until the *w*/*w* ratio reached 30, indicating that PEI 2 kDa has lower cytotoxicity and higher transfection efficiency to some extent. Therefore, polycationic gene vector was constructed based on low-molecular-weight PEI, which could not only ensure lower cytotoxicity but also improve transfection efficiency, so as to effectively deliver p53 gene. Compared with PEI 2 kDa and PEI 25 kDa, CUR-PEI-K14/DNA showed obvious green fluorescent protein expression at the *w*/*w* ratios of 5–30, and the transfection efficiency of the complexes first rose and then declined as the *w*/*w* ratio increased. The transfection efficiency of CUR-PEI-K14-3/DNA reached a peak at *w*/*w* ratio of 15, and clear and dense green fluorescence could be seen in *w*/*w* = 10 of CUR-PEI-K14-6/DNA. These results showed that the transfection efficiency of CUR-PEI-K14/DNA complexes was higher than that of PEI 2 kDa/DNA and PEI 25 kDa/DNA in SKOV3-DDP cells, confirming that CUR-PEI-K14 polymer had excellent transfection performance.

The *pGL3-Control* was chosen to further evaluate in vitro transfection efficiency of CUR-PEI-K14 in SKOV3-DDP cells. As shown in Figure 6b, the transfection efficiency of CUR-PEI-K14/DNA increased at first and then decreased, which was consistent with the green fluorescent protein expression results. The transfection efficiency of the complexes-modified K14 was significantly higher than that of unmodified K14, indicating the introduction of K14 could increase the delivery of DNA. The introduction of tLyP-1 and NLS made the co-delivery system enter into the cell better through the NRP and pass through the nuclear membrane better, which was confirmed in our previous study [24]. But the large proportion of K14 reduced the transfection efficiency, which may be related to the partial charge shielding of PEI by K14. The transfection efficiency of CUR-PEI-K14-3/DNA in SKOV3-DDP cells was significantly higher than that of PEI 2 kDa and PEI 25 kDa under the same *w*/*w* ratio, and reached the highest when *w*/*w* was 10, which meant that p53 would have a better transfection.

### 2.6. Cytotoxicity of CUR-PEI-K14

**Figure 7:**
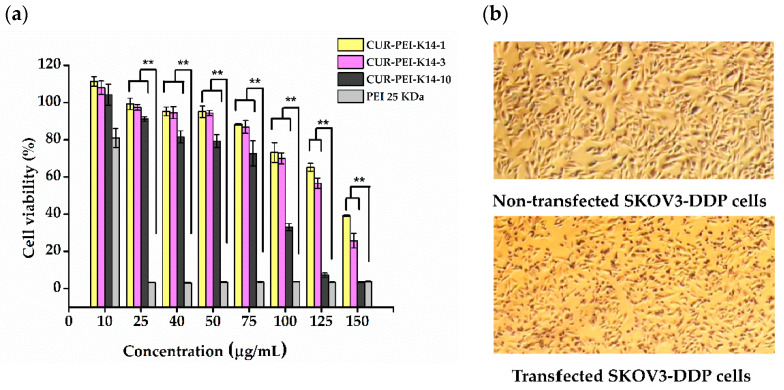
(**a**) Cytotoxicity of CUR-PEI-K14 and PEI 25 kDa at various concentrations in SKOV3-DDP cell lines using the 3-(4,5-dimethyl-2-thiazolyl)-2,5-diphenyl-2-*H*-tetrazolium bromide (MTT) assay. (**b**) Representative microscopic images of nontransfected SKOV3-DDP and transfected SKOV3-DDP cells. Each data point represents the mean ± standard deviation (*n* = 6, ** *P* < 0.01).

Figure 7 shows that the cell viability of SKOV3-DDP cells gradually decreased with increasing polymer concentration. This phenomenon indicated that the cytotoxicity of CUR-PEI-K14 was dose-dependent. At a low concentration (≤75 μg/mL), the cell viability was more than 80% and there was no significant difference among CUR-PEI-K14-1, CUR-PEI-K14-3, and CUR-PEI-K14-10. However, the cytotoxicity of CUR-PEI-K14-10 was significantly higher than that of CUR-PEI-K14-1 and CUR-PEI-K14-3 at a high concentration (≥100 μg/mL). It was worth noting that when PEI 25 kDa concentration was ≥25 μg/mL, the cell viability was close to 0, while CUR-PEI-K14-1 and CUR-PEI-K14-3 at the same concentration both showed high cell viability, which indicated that CUR-PEI-K14 had good safety for in vivo delivery. Moreover, it could be seen from the cell morphology before and after transfection that the changes in cell morphology were not obvious, which also reflected that the vector was less toxic.

### 2.7. Enhancing the Sensitivity of SKOV3-DDP Cells to Cisplatin by CUR-PEI-K14/p53

**Figure 8:**
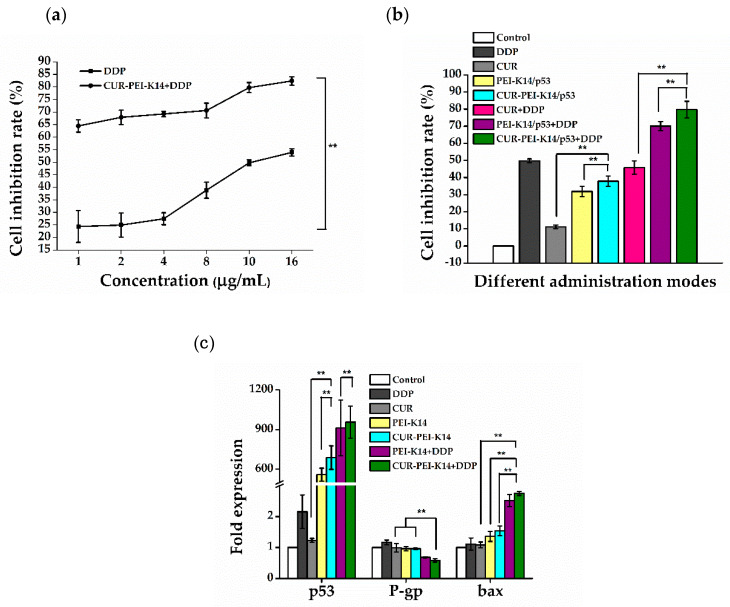
(**a**) Sensitizing effect of CUR-PEI-K14/p53 on DDP in SKOV3-DDP cells. (**b**) The cell inhibition rate of SKOV3-DDP cells in different administration modes. (**c**) Expression of p53, P-gp, and bax mRNA in SKOV3-DDP cells in different administration modes. Each data point represents the mean ± standard deviation (*n* = 6, ** *P* < 0.01).

As can be seen from Figure 8a, the cell inhibition rate increased with the increase of DDP concentration, and the half-inhibitory concentration (IC_50_) was about 10 μg/mL. After CUR-PEI-K14/p53 treatment, the inhibition rate of DDP on SKOV3-DDP cells at all concentrations was significantly increased, especially at the low concentration of DDP (1 μg/mL), which increased by about 3 times from 24.35% to 64.45%, even exceeding the cell inhibition rate (49.76%) of 10 μg/mL DDP alone. At this time, the IC_50_ of DDP decreased to less than 1 μg/mL, indicating that CUR-PEI-K14/p53 significantly increased the sensitivity of drug-resistant cells to DDP. The synergistic effect of CUR and p53 in enhancing the sensitivity of SKOV3-DDP cells to DDP was further studied by fixing DDP concentration.

As shown in Figure 8b, after treatment of SKOV3-DDP cells with DDP, CUR, p53, and combination groups for 24 h, the cell inhibition rate of each group was significantly different. CUR and p53 alone had a slight inhibitory effect on the growth of SKOV3-DDP cells, which were much weaker than 10 μg/mL of DDP (inhibition rate 49.76%) even when used in combination. Under the condition of fixed DDP concentration, the cell inhibition rate of DDP combined with CUR-PEI-K14/p53 increased significantly (up to 79.73%), indicating that the co-delivery of CUR and p53 was helpful to enhance the cell sensitivity to DDP. In addition, the combined application of DDP and CUR did not increase the inhibition effect of DDP, while the combined application of DDP and p53 increased the inhibition effect of DDP, but the inhibition effect of DDP reached the highest after DDP combined with CUR and p53 (CUR-PEI-K14/p53 + DDP), indicating that CUR and p53 synergistically enhanced the sensitivity of DDP to SKOV3-DDP cells.

Figure 8c shows the expressions of p53, P-gp, and bax mRNA in SKOV3-DDP cells in different treatment groups. Compared with the control group, there was no significant difference in p53 expression of DDP or CUR treated cells. After treatment with PEI-K14/p53 or CUR-PEI-K14/p53, the expression of p53 was significantly increased, indicating that p53 had been successfully transported into cells and started to be expressed. Interestingly, DDP alone could not induce the expression of p53 mRNA, but combined treatment with CUR-PEI-K14/p53 promoted the expression of p53 mRNA (higher than CUR-PEI-K14/p53 alone), and p53 had a tumor cell inhibitory effect, which meant increased apoptosis, suggesting that CUR-PEI-K14/p53 effectively enhanced the inhibitory effect of DDP on SKOV3-DDP cells.

The stimulation of DDP could increase the expression of P-gp mRNA in SKOV3-DDP cells, which enhanced the resistance of cells to DDP. Other groups could downregulate the expression of P-gp mRNA, which could inhibit the efflux of cytotoxic drugs such as DDP, increase the intracellular drug concentration, and enhance the cytotoxic effect. In PEI-K14/p53 + DDP and CUR-PEI-K14/p53 + DDP groups, combined use of CUR and p53 was obviously superior to that of p53 alone, which confirmed the inhibition of CUR on P-gp and the expression level of P-gp mRNA in CUR-PEI-K14/p53 + DDP group was the lowest, which may be related to the synergism of CUR and p53 in reducing the expression of P-gp mRNA.

Compared with the control group, the expression of bax mRNA increased in other groups. The increase in CUR group was not significant, indicating that CUR had slight effect on promoting apoptosis. However, when combined with p53, the expression level of CUR-PEI-K14/p53 group was higher than that of PEI-K14/p53, suggesting that CUR and p53 had a synergistic effect in upregulation of bax mRNA. It was worth noting that although DDP could promote the expression of bax mRNA, it increased significantly when combined with PEI-K14/p53 and CUR-PEI-K14/p53, indicating that p53 and CUR could significantly enhance the sensitivity of SKOV3-DDP cells to DDP.

## 3. Materials and Methods

### 3.1. Materials and Cell Lines

The 4-(*N*-Maleimidomethyl)cyclohexanecarboxylic acid *N*-hydroxysuccinimide ester (SMCC), polyethyleneimine (branched PEI, molecular weight 2000 Da), 3-(4,5-dimethylthiazol-2-yl)-2,5-diphenyl tetrazolium bromide (MTT), 3,8-Diamino-5-ethyl-6-phenylphenanthridinium bromide (EB), glutaric anhydride (95%), heparin, and loading buffer were obtained from Sigma-Aldrich (St. Louis., MO, USA). The 0.1 mol/L (M) phosphate buffer saline (PBS) was purchased from Biosharp (Shanghai, China). Dimethylsulfoxide (DMSO), ethyl acetate (EtOAc) and tetrahydrofuran (THF) were obtained from Sinopharm Chemical Reagent Co., Ltd. (Shanghai, China). *N*,*N*-Dimethylformamide (DMF) was purchased from Adamas-beta (Shanghai, China). Triethylamine (Et_3_N) was purchased from Macklin Biochemical Co., Ltd. (Shanghai, China). Cisplatin (DDP) was purchased from TargetMol (Boston, MA, USA). Curcumin (CUR) and 4-dimethylaminopyridine (DMAP) were purchased from TCL (Tokyo, Japan). The 1-(3-Dimethylaminopropyl)-3-ethylcarbodiimide hydrochloride (EDC) and *N*-Hydroxysuccinimide (NHS) were obtained from Aladdin Reagent Co., Ltd. (Shanghai, China). A dialysis bag (MW cut-off 500) was purchased from Shanghai Gene Star Co., Ltd. (Shanghai, China). Amicon Ultra-0.5 Ultracel-3 membrane, 3 kDa was obtained from Millipore (Bedford, MA, USA). Roswell Park Memorial Institute (RPMI) 1640 culture medium, phosphate buffer saline (PBS), and fetal bovine serum (FBS) were purchased from Gibco (Paisley, Scotland, UK). TRIzol reagent was purchased from Thermo Fisher (Waltham, MA, USA). Talent qPCR PreMix (SYBR Green) and Fasting Real-Time (RT) Kit (with gDNase) was purchased from Tiangen Biotech Co., Ltd. (Beijing, China). The peptides of CKKKRKCGNKRTRC and CPLGLAG were synthesized by GL Biochem (Shanghai, China).

The *pEGFP-N2* and *pGL3-Control* plasmids used for in vitro transfection assays were stored in the laboratory, and the recombinant p53 plasmids were commissioned by Shanghai Chunguang Biotechnology Co., Ltd. (Shanghai, China) to synthesize. The plasmid was amplified using *E. coli* TOP 10 (Shanghai Chunguang Biotechnology Co., Ltd., Shanghai, China), and prepared using Qiangen End-free plasmid Mega Kit (Qiangen, Hilden, Germany). The protein concentration was determined by bicinchoninic acid (BCA) protein assay kit (Bioteke Corporation, Beijing, China).

SKOV3-DDP cells were obtained from Shanghai Chunguang Biotechnology Co., Ltd. (Shanghai, China). The cells were cultured in RPMI 1640 medium (Gibco, Scotland, UK) with 15% (*v*/*v*) fetal bovine serum (FBS) (Gibco, Scotland, UK). To maintain resistance, cells were cultured in medium containing 0.2 μg/mL DDP.

### 3.2. Synthesis and Characterization of CUR-PEI-K14

#### 3.2.1. Preparation of PEI-K14

First, the bifunctional peptide K14 was synthesized using solid-phase method. Then, PEI was cross-linked with K14 using SMCC as cross-linker. PEI and SMCC were dissolved in 0.1 M PBS and DMSO, respectively. Then, the appropriate amount of SMCC solution (3.33 mg/mL) was added to the PEI solution (10 mg/mL) at a molar ratio of 1:3, 1:1, 2:1, and 10:3. The reaction mixture was stirred for 30 min. The unreacted cross-linker was removed by gel chromatography (Pharmacia, Piscataway, NJ, USA). K14 (10 mg/mL in 0.1 M PBS) was then added dropwise to the PEI solution at a molar ratio of 1:1, 3:1, 6:1, and 10:1. The reaction mixture was stirred for 12 h at 4 °C and then lyophilized after the nonreacted K14 was removed by ultrafiltration. The PEI-K14 were obtained and named PEI-K14-1, PEI-K14-3, PEI-K14-6, and PEI-K14-10, respectively.

PEI and PEI-K14 were characterized by ^1^H-NMR. PEI and PEI-K14 (10 mg) were dissolved in 0.6 mL of deuterium oxide (D_2_O) in an NMR tube, respectively, and the ^1^H-NMR spectra were recorded using a Varian 300-MHz spectrometer (Varian Medical Systems, Palo Alto, CA, USA) at room temperature.

#### 3.2.2. Preparation of CUR-PEI-K14

First, the CPLGLAG peptide was synthesized using solid-phase method. Then, CUR (1.00 g) and DMAP (56 mg) were dissolved in THF (50 mL), and Et_3_N (0.665 mL) was added to the solution. Then, glutaric anhydride (95%) (0.3425 g) was dissolved in THF (5 mL) and added to CUR solution. The reaction mixture was stirred overnight. After the solvent was removed, EtOAc (55 mL) and 1 M HCl (15 mL) were added. The mixture was stirred for 10 min. The organic phase was separated and extracted with EtOAc. The solvent was removed and dried. The product was purified by column chromatography and eluted with CH_2_Cl_2_:MeOH (95:5) to obtain CUR-COOH [43].

CUR-COOH (5 mg), NHS (3.5 mg), and EDC (3.5 mg) were dissolved in anhydrous DMF (3 mL). The mixture solution was stirred in ice bath for 6 h. The CPLGLAG peptide (5 mg) was dissolved in THF (5 mL) and added to the above-mentioned reaction solution. The reaction mixture was stirred for 48 h at room temperature and then dialyzed in DMSO for 48 h using a dialysis bag with a cut-off molecular weight of 500. Then, the reaction mixture was dialyzed in deionized water for another 48 h. After the end of dialysis, CUR-CPLGLAG was obtained through lyophilizing.

The appropriate amount of SMCC solution (3.33 mg/mL) was added to the PEI-K14 solution (10 mg/mL) at a molar ratio of 1:1. The reaction mixture was stirred for 30 min. The unreacted SMCC was removed. The CUR-CPLGLAG solution (10 mg/mL) was then added to PEI-K14 solution (10 mg/mL) at a molar ratio of 6:1. The reaction mixture was stirred for 12 h at 4 °C and then lyophilized after the nonreacted CUR-CPLGLAG was removed by ultrafiltration. The CUR-PEI-K14 were obtained and named CUR-PEI-K14-1, CUR-PEI-K14-3, CUR-PEI-K14-6, and CUR-PEI-K14-10, respectively. CUR-PEI-K14 was characterized by ^1^H-NMR (Varian Medical Systems, Palo Alto, CA, USA).

### 3.3. Condensation of Plasmid DNA by CUR-PEI-K14

At first, 10 μL of DNA solution at a concentration of 50 μg/mL was added into a 1.5-mL tube. Then, 10 μL of CUR-PEI-K14 solution at different concentrations (5, 10, 20, 30, 40, 50, 60, 70, and 80 μg/mL) were added to DNA solutions, respectively. The mixtures were allowed to stand at 4 °C for 30 min. Complexes with different *w*/*w* ratios (0.1, 0.2, 0.4, 0.6, 0.8, 1.0, 1.2 1.4, 1.6) were obtained. Then, 1 μL of 10× loading buffer was added to complex solution. The sample was electrophoresed on 1% agarose gel for about 30 min at 120 V. At last, the gel was stained with ethidium bromide (0.5 μg/mL) for about 15 min and illuminated on a UV illuminator (Bio-Rad, Hercules, CA, USA) to show the DNA bands.

### 3.4. Particle Size, Zeta Potential, and Morphology of CUR-PEI-K14

CUR-PEI-K14/DNA complexes at desired *w*/*w* ratios were prepared and incubated at 4 °C for 30 min. Then, the particle size and zeta potential were measured by an electrophoretic light-scattering spectrophotometer (Zetasizer Nano ZS90, MAN0317 Issue 5.0; Malvern Instruments Ltd., Malvern, UK). The prepared complexes were dropped to the center of the copper grid. After samples were dried, the morphological characteristics of complexes were observed by scanning electron microscopy (Tescan Mira3, Shanghai, China).

### 3.5. Resistance of CUR-PEI-K14/DNA Complex to Heparin Dissociation and Serum

Ten μL of CUR-PEI-K14/DNA solution was added to a 1.5-mL tube. Different concentrations of sodium heparin solution or fetal bovine serum (FBS) were added to the complex solution and incubated at 37 °C for 60 min. Then, electrophoresis was performed to examine the protection of CUR-PEI-K14 on pDNA from dissociation by sodium heparin or serum.

### 3.6. In Vitro Transfection

The *pEGFP-N2* and *pGL3-Control* were used as report genes to measure the transfection efficiency of CUR-PEI-K14 in SKOV3-DDP cells. First, cells were inoculated in a 24-well plate at a density of 1 × 10^5^ cells per well, and incubated to reach 80% confluence. Then, 100 μL of DNA complexes’ solution at different *w*/*w* ratios and 400 μL of RPMI 1640 medium were added to a 24-well plate and incubated at 37 °C with 5% CO_2_ for 4 h. After the old medium was replaced with fresh medium containing 15% FBS, cells were incubated for another 48 h. Afterwards, the *pEGFP-N2* expression was observed with an inverted fluorescent microscope (AE-31; Motic Corporation, Wetzlar, Germany).

The luciferase assay was performed according to the manufacturer’s instructions. After the cells were washed with PBS, 100 μL of cell culture lysing agent was added and shaken for 30 min. The luciferase activity was examined with a luminometer (Turner Designs Luminometer Model TD-20/20; Promega Corp., Madison, WI, USA). The protein contents were measured by a bicinchoninic acid protein assay kit (PP1001; Bioteke Corporation, Beijing, China). The transfection efficiency for the *pGL3-Control* was calculated according to the relative light unit (RLU) against the corresponding protein content.

### 3.7. MTT Assay

At first, SKOV3-DDP cells were inoculated in a 96-well plate at a density of 5 × 10^3^ cells per well, and incubated to reach 80% confluence. Then, the growth medium was replaced with 200 μL of RPMI 1640 medium containing different polymers at various concentrations to measure the cytotoxicity. The cells were incubated for 72 h. Then, the old medium was replaced with 20 μL MTT (5 mg/mL) and 180 μL serum-containing medium and left to incubate for another 4 h. At last, the MTT solutions were removed and 150 μL of DMSO were added to dissolve the formazan crystals. The absorbance value was measured in a microplate reader (Model 680; Bio-Rad, Hercules, CA, USA) at 570 nm. The same method was also used to evaluate the effect of CUR-PEI-K14/p53 on enhancing the sensitivity of SKOV3-DDP cells to DDP. In the combined treatment group, the cells were first transfected and then exposed to DDP. The cell viability (%) and cell inhibition rate (%) were calculated according to the following equations:

Cell viability (%) = (A_test_/A_control_) × 100 (1)

Cell inhibition rate (%) = (1 − A_test_/A_control_) × 100 (2)

where A_test_ is the absorbance value for treated cells and A_control_ is the absorbance value for untreated cells (mean ± standard deviation, *n* = 6).

### 3.8. Real-Time Quantitative RT-PCR

RT-PCR was used to analyze the expression of p53, P-gp, and bax mRNA. SKOV3-DDP cells were seeded in 6-well plates at a density of 1 × 10^6^ cells/well and incubated for 24 h. The cells were treated with different administration modes. After completeness of incubation, cells were collected to extract total RNA using TRIzol (Thermo Fisher, Waltham, MA, USA), and then reverse-transcribed into complementary DNA (cDNA) using Fasting RT Kit (with gDNase) (Tiangen Biotech Co., Ltd., Beijing, China). The obtained cDNA was used as the template for PCR reactions. RT-PCR was performed using the Talent qPCR PreMix (SYBR Green) (Tiangen Biotech Co., Ltd., Beijing, China) according to the manufacturer’s protocol. PCR reactions were run on an ABI StepOnePlus Real-Time PCR System (ABI, Foster City, CA, USA). PCR was performed for 60 s at 95 °C, then 5 s at 95 °C and 40 s at 60 °C, 40 cycles.

The forward and reverse sets of several primer pairs are as follows: GAPDH (5’-GTG GAG TCC ACT GGC GTC TT-3, 5’-GTG CAG GAG GCA TTG CTG AT-3’); p53 (5’-CCA TGA GCG CTG CTC AGA T-3’, 5’-AGG GCA CCA CCA CAC TAT GTC-3’); P-gp (5’-GGT GCT GGT TGC TGC TTA CA-3’, 5’-ACA TCG TGC ACA TCA AAC CA-3’); bax (5’-CGA GTG GCA GCT GAC ATG TT-3’, 5’-GAT CAG TTC CGG CAC CTT G-3’).

### 3.9. Statistical Analysis

The data were expressed as means ± standard deviation. The statistical significance was evaluated using one-way analysis of variance. A *p*-value < 0.05 was set statistically significant in all the tests.

## 4. Conclusions

At present, enhancing the sensitivity of drug-resistant cells to platinum is still an important topic of platinum chemotherapy for ovarian cancer, which is of practical significance to prolong the survival time of advanced patients. In this work, we developed a co-delivery system, CUR-PEI-K14/p53, by cross-linking LMW PEI with a bifunctional peptide K14, and further coupling with CUR through CPLGIAG peptide, and at last self-assembled with p53. Various physicochemical methods were used to prove that the constructed CUR-PEI-K14 could achieve the assumption to deliver p53 and CUR. The results confirmed that CUR-PEI-K14 had great ability to condense DNA and suitable physicochemical properties to co-deliver DNA and drug. Furthermore, the co-delivery system showed higher gene transfection efficiency and lower cytotoxicity compared with PEI 25 kDa in SKOV3-DDP cell lines. Finally, it was confirmed by MTT and RT-PCR that CUR-PEI-K14/p53 could efficiently deliver p53 gene, and significantly enhanced the inhibitory effect of DDP on SKOV3-DDP cells by downregulating the expression of P-gp and upregulating the expression of p53 and bax. In conclusion, this co-delivery system can be used as an effective strategy to enhance the sensitivity of drug-resistant ovarian cancer cells to DDP. However, it is worth investigating whether CUR-PEI-K14 can show excellent characteristics such as targeting, low cytotoxicity, and high transfection efficiency in vivo. In addition, the sensitizing effect of CUR-PEI-K14/p53 needs to be studied in animal models. We are currently conducting more comprehensive studies with the aim to answer these questions.

## Supplementary Materials

The following are available online, Figure S1: SEM image of CUR-PEI-K14 polymers.
