# Supplementary material for: A Co-Delivery System of Curcumin and p53 for Enhancing the Sensitivity of Drug-Resistant Ovarian Cancer Cells to Cisplatin
Source: Molecules. 2020 Jun 4;25(11):2621. doi: 10.3390/molecules25112621 (PMC7321199; doi:10.3390/molecules25112621)
Supplement: Supplementary file 1 [file molecules-25-02621-s001.pdf]

SUPPLEMENTARY MATERIALS

A co-delivery system of curcumin and p53 for enhancing the sensitivity of drug-resistant ovarian cancer cells to cisplatin

Xinli Guo, Zhou Fang, Min Zhang, Deyu Yang, Shuyue Wang and Kehai Liu *

Department of Biopharmaceutics, School of Food Science and Technology, Shanghai Ocean University, Shanghai 201306, China; gxllps@163.com (X. G.); fangzhou0117@163.com (Z.F.); 626250257@qq.com (M.Z.); 2651999212@qq.com (D.Y.); 534558644@qq.com (S.W.)

* Correspondence: khliu@shou.edu.cn; Tel.: +86-216-190-0388 (K.L.)

SUPPLEMENTARY MATERIALS CONTENT

Figure S1. SEM image of CUR-PEI-K14 polymers.

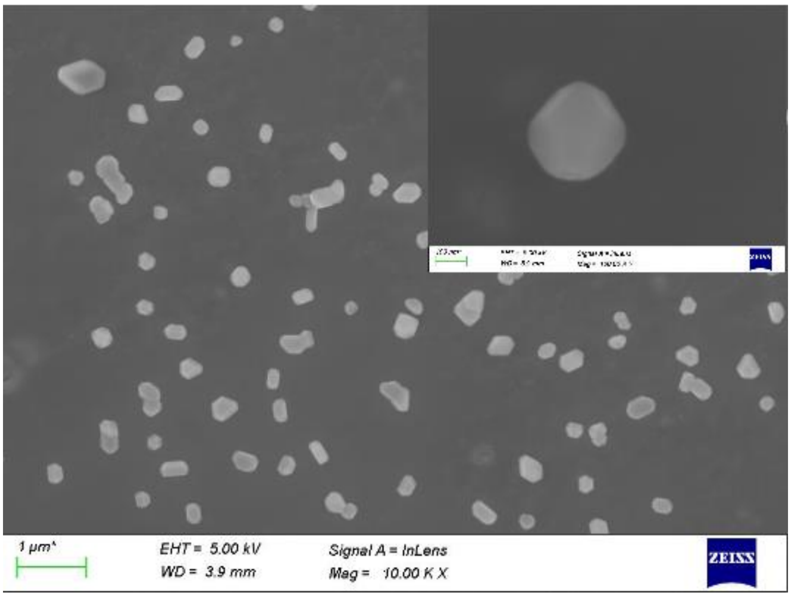

Figure S1. SEM image of CUR-PEI-K14 polymers.
